# Supplementary material for: Spontaneous Myocarditis in Mice Predisposed to Autoimmune Disease: Including Vaccination-Induced Onset
Source: Biomedicines. 2022 Jun 18;10(6):1443. doi: 10.3390/biomedicines10061443 (PMC9220133; doi:10.3390/biomedicines10061443)
Supplement: Supplementary file 1 [file biomedicines-10-01443-s001.zip › Supplementary Figures.pdf]

Supplementary Data sets

Spontaneous myocarditis in mice predisposed to autoimmune disease: Including Vaccination-induced onset

Takuma Hayashi, Motoki Ichikawa, Ikuo Konishi.

Supplementary material

1. Materials and Methods

1.1. Animals

D2.129P2 (B6)-*Nfκb1* homozygote mice were purchased from JAX ® Mice and Services at The Jackson Laboratory (Bar Harbor, ME, USA). Non-obcsc diabctic (NOD)/ShiLtJ mice were purchased from CLEA Japan, Inc. (Meguro, Tokyo, Japan). F15 NOD *Nfκb1* heterozygote was created by back crossing D2.129P2 (B6)-*Nfκb1* homozygote male mice with NOD/ShiLtJ female mice or D2.129P2 (B6)-*Nfκb1* homozygote female mice with NOD/ShiLtJ male mice (F15 means backcrossed more than 15 times). In addition, we crossed F15 NOD *Nfκb1* heterozygote males and females to create F15 NOD *Nfκb1* homozygote. All experiments were performed using age and sex-matched groups. Mice were maintained in specific pathogen-free (SPF) facilities at Shinshu University (Matsumoto, Nagano, Japan). All of the study protocols were approved by the Animal Care and Use Committee of the Shinshu University and National Hospital Organization Kyoto Medical Center (Approved number 2016-3, 2019-03). Experiments were performed according to the official rules formulated in the Japanese law on the care and use of experimental animals.

The genotyping protocol(s) presented here have been optimized for reagents and conditions used by The Jackson Laboratory (JAX). The genotyping analyses were performed using primer sets as followings, Common 5'-GCA AAC CTG GGA ATA CTT CAT GTGACT AAG-3', wild type 5'-ATA GGC AAG GTC AGA ATG CAC CAGAAG TCC-3', mutant 5'-AAA TGT GTC AGT TTC ATA GCC TGAAGA ACG-3'. The detail of genotyping protocol by PCR is described in following JAX website, https://www.jax.org/Protocol?stockNumber=002849&protocolID=19175

1.2. M-mode echocardiography examination.

To determine the disease severity of myocarditis, M-mode echocardiography measurements obtained from mice was performed by SonoSite M using standard procedure (FUJIFILM SonoSite, Inc., Minato-ku, Tokyo, Japan). Briefly, mice were anesthetized with ether and examined by M-mode echocardiography.

1.3. Flow cytometry.

One million splenocytes obtained from D2.129P2 (B6)-*Nfκb1* homozygote mice, NOD/ShiLtJ mice, and F15 NOD *Nfκb1* heterozygote were suspended in biotin-free RPMI containing 0.1% azide and 3% FCS and surface stained in 96-well plates with the 10–3.6 PE (anti-I-Ag7) (BD PharMingen, Franklin Lakes, NJ, USA), which is class II major histocompatibility complex (MHC) haplotype for NOD/ShiLtJ mice. The splenocytes were washed no fewer than two times before the addition of the secondary reagent. All samples were analyzed on a FACSCalibur flow cytometer (Becton Dickinson, Mountain View, CA, USA) using CellQuest software (Becton Dickinson).

		MHC Class I			MHC Class II		MHC Class Ib				
Mouse Strains	MHC Haplotype	H-2K	H-2D	H-2L	I-A	I-E	Qa-2	Qa-1	CD45 (Ly-5)	Thy-1 (CD90)	NK1.1
NOD	g7	d	b	blank	g7	null	a			2	-

https://tools.thermofisher.com/content/sfs/brochures/Mouse_Haplotype_Table.pdf

Appendix 1: g7 as genetical marker for NOD/ShiLtJ mouse.

1.4. Staining and Immunohistochemistry (IHC).

IHC staining for Cluster of Differentiation 3 (CD3) was performed on serial heart sections (horizontal section) and pancreatic sections obtained from F15 NOD *Nfκb1* wild type mice, F15 NOD *Nfκb1* heterozygote mice, and F15 NOD *Nfκb1* homozygote mice (Supplementary material materials and methods *1.1*). The monoclonal antibody for mouse CD3 (17A2, 1:200) was purchased from Thermo Fisher Scientific (Waltham, MA, USA). IHC was performed using the avidin–biotin complex method, as described previously. Hematoxylin and Eosin (H.E.) staining was performed by standard procedure. Briefly, one representative 5-mm tissue section was cut from a paraffin-embedded sample of heart and pancreatic tissues obtained from F15 NOD *Nfκb1* wild type mice, F15 NOD *Nfκb1* heterozygote mice, and F15-NOD *Nfκb1* homozygote mice.

Next, the sections were incubated with a biotinylated secondary antibody (Dako, DK-2600 Glostrup, Denmark) and then incubated with a streptavidin complex (Dako). The completed reaction was developed by 3, 39-diaminobenzidine, and the slide was counterstained with hematoxylin. Normal myometrium portions in the specimens were positive controls. The negative controls comprised tissue sections incubated with normal rabbit IgG instead of the primary antibody. The expression of CD3 is indicated by brown 3,3'-Diaminobenzidine, tetrahydrochloride (DAB) staining. Normal rabbit antiserum was a negative control for the primary antibody. The entire brown DAB-stained tissue was scanned with a digital microscope BZ-X800 (Keyence, Osaka, Osaka, Japan). Hearts and pancreas were then removed from mice, fixed in 4% formalin, and paraffin embedded. Sections were obtained at eight different levels and stained with H.E. by standard procedure. Brown dots indicate the lymphocytes expressing murine CD3. Shinshu University approved these experiments according to internal guidelines (approval no. M192).

The sections used for Immunohistochemistry (IHC) staining are the slices obtained from cross sections cut in a horizontal section at the position of the mouse heart (4.5 mm from the top) shown in the figure below.

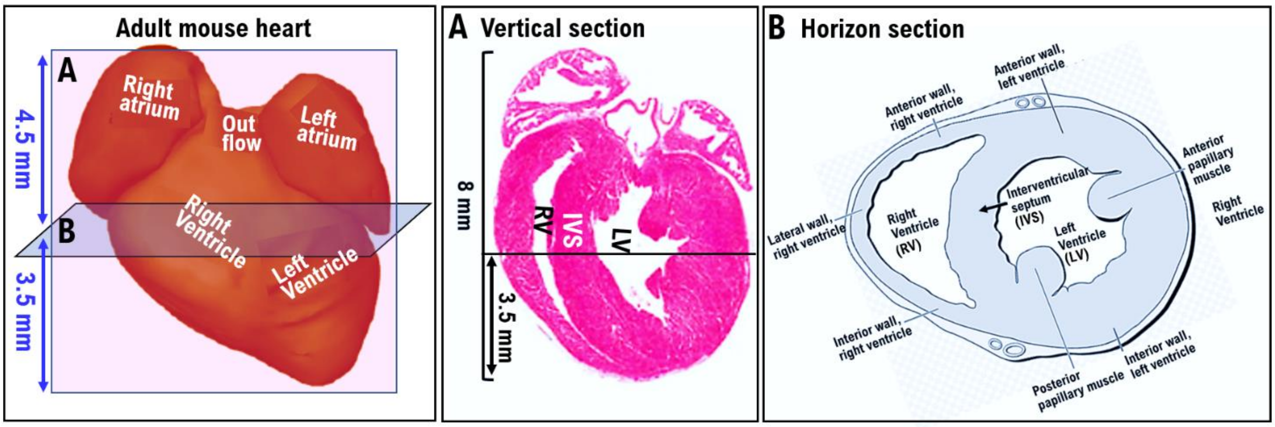

Appendix 2: Position of cross section of slice for immunohistochemical staining and pathological examination.

1.5. Vaccine immunization.

Intramuscular injection with 20 μL or 50 μL of influenza vaccine (influenza HA vaccine "KMB"; KM Biologics Co. Ltd., Kumamoto-city, Kumamoto, Japan) or 50 μL of hepatitis B virus (HBV) vaccine (Bimmugen; KM Biologics Co. Ltd., Kumamoto-city, Kumamoto, Japan), 50 μL of CERVARIX (2vHPV vaccine: GlaxoSmithKline plc, Borough of Hounslow London, UK), 50 μL of GARDASIL (4vHPV vaccine: MSD K.K. Chiyoda, Tokyo, Japan) or Phosphate Buffered Saline (PBS) (FUJIFILM Wako Pure Chemical Corporation, Osaka-city, Osaka, Japan) as immunogen at quadriceps femoris muscle of 10 weeks old 129P2(B6)-*Nfkb1* wild type mice, heterozygote mice, homozygote mice and *F15 NOD Nfkb1* wild type mice, heterozygote mice, and NOD/ShiLtj mice were performed for immunological studies including cardiological studies, and allergy assay. At 30

days after 1st dose immunization of influenza HA vaccine, Bimmugen, CERVARIX, GARDASIL or PBS, the mice received second doses of influenza HA vaccine, Bimmugen, CERVARIX, GARDASIL or PBS. Each vaccine was diluted twice with PBS and inoculated into the mice. We purchased influenza HA vaccine and Bimmugen from KM Biologics Co. Ltd., and CERVARIX and GARDASIL from Japan Vaccine Co., Ltd. (Chiyoda, Tokyo, Japan).

1.6. cardiac troponin T (cTnT) ELISA and myocarditis scoring.

To correlate serum cardiac troponin T (cTnT) elevations with the presence and severity of myocarditis, briefly, mice were anesthetized with ether and bled retro-orbitally or alternatively, blood was obtained by cardiac puncture at the time of dissection (Supplementary data excel file). Serum samples were stored individually at −70°C until use. Immediately after bleeding, mice were killed by cervical dislocation. Hearts were then removed, fixed in 4% formalin, and paraffin embedded. Sections were obtained at eight different levels and stained with H.E.. The diagnosis of myocarditis was established by the presence of an inflammatory cell infiltrate and myocyte damage. The disease severity was determined according to a previously described scoring system ranging from 0 to 4 (1 corresponds to infiltration of ≤5% of at least one histological cross section; 2, 5% to 10%; 3, 10% to 20%; and 4, >20%). Differences in disease severity were analyzed by ANOVA for multiple-sample comparisons (Bonferroni).

cTnT ELISA: Serum cardiac troponin T (cTnT) levels were determined with mouse cTnT ELISA Kit (CUSABIO TECHNOLOGY LLC, Houston, TX, USA) according to the manufacturer’s instructions. This sandwich ELISA system is based on two monoclonal Antibodies, namely, a biotinylated cTnT–specific capture antibody (CSB-PA024016HA01MO, CUSABIO TECHNOLOGY LLC, Houston, TX, USA) that binds to streptavidin-coated plastic tubes and a horseradish peroxidase–labeled detection antibody.

1.7. Multiplex cytokine bead array assay and mouse IgG assay

To collect serum for measuring of levels of serum cytokines, mice were anesthetized with ether and bled retro-orbitally or alternatively at 3 days after immunizations of 4 types of vaccines. Cytokine and chemokine levels were measured in mouse serum (20× diluted) using the Mouse Cytokine Array Panel A (Catalog Number ARY006; R&D Systems, Inc., Minneapolis, MN, USA) based on the manufacturer's instructions. Serums were collected from each mouse at the time of dissection (Supplementary data in the excel file).

To collect serum for measuring of levels of serum cytokines, mice were anesthetized with ether and bled retro-orbitally or alternatively at 3 days after immunizations of 4 types of vaccines. The levels of mouse IgG were measured in mouse serum using the Mouse IgG ELISA Kit (ab151276) (Catalog Number ab151276; Abcam plc. Cambridge Biomedical Campus, Cambridge, CB2 0AX, UK) based on the manufacturer's instructions.

1.8. Naïve CD8$^+$ T Cell Isolation and Proliferation assay for CD8$^+$ T cells.

Splenocytes from the mice were harvested and depleted of red blood cells. Then, CD8$^+$ T cells were sorted using a magnetic-activated cell sorting kit (CD8$^+$ T Cell Isolation Kit II, Miltenyi Biotech, Bergisch Gladbach Germany) according to the manufacturer's protocol as previously described. The isolated CD8$^+$ T cells were resuspended in PBS containing 2% FBS, CD8$^+$ T cells at a concentration of 1x10^6 cells/ml were co-cultured with 100 µl of PBS containing a range of cardiac myosin heavy chain-α aa334-352 peptide (Cosmo Bio Co., Ltd., Koto-ku, Tokyo, Japan) or containing a range of Influenza A HA (46-54) Peptide (FMYSDFHFI) (M&S TechnoSystems, Inc., Osaka-city, Osaka, Japan) (5 µg/mL or 10 µg/mL), containing a range of mouse interleukin-2 (IL-2) concentrations (10 IU/mL or 50 IU/mL), or a range of mouse IL-12 concentrations (10 ng/mL or 20 ng/mL) (PeproTech, Shanghai, China), for a period of 48 hours. The cultured CD8$^+$ T cells were harvested, and the proliferation of CD8$^+$ T cells was evaluated using the BrdU Cell Proliferation ELISA kit (cat. no. ab126556; Abcam, Cambridge Biomedical Campus, Cambridge, MA, USA) based on the

manufacturer's instructions. The supernatant was harvested and analyzed for the release of Interferon gamma (IFN-γ).

After incubation for a period of 48 hours, the culture supernatants were collected for cytokine detection. IFN-γ levels in the culture supernatants were measured by enzyme-linked immunosorbent assays (ELISAs) using a commercial kit (R&D Systems, Inc., Minneapolis, MN, USA) based on the manufacturer's instructions.

1.9. Allergy assay
Immunoglobulin E (IgE) levels were measured in mouse serum (10 × diluted) using OpIEATM set mouse IgE (BD PharMingen, Lakes, NJ, USA) based on the manufacturer's instructions. Ten weeks-old BALB/c mice (Japan Clear, Meguro-ku, Tokyo, Japan) were intraperitoneally administered 100 μL of a solution containing Formalin treated Pseudomonas pertucinogena (FTPP) suspension (80 μg/Body) with Mite Extract DF (50 μg/Body) as experimental allergy model mouse, the mice were intraperitoneally second doses administered 100 μL of FTPP suspension and Mite Extract DF at 2 weeks after first injection. Serums were collected from each treated BALB/c mouse at 2 weeks after second doses injection. The standard value of IgE in adult is less than 170 IU/mL, probably IgE of babyhood at early stage is 5.0-10.0 IU/mL. FTPP suspension and Mite Extract DF were purchased from LSL Co. Ltd., (Kohto-ku, Tokyo, Japan).

1.10. Statistical Analysis
All data are expressed as mean ± SEM. Normality was verified using the Shapiro-Wilk test. Statistical analyses were performed using an unpaired two-tailed *t* test or Mann-Whitney *U* test for comparison of two groups. For multiple comparisons, one-way analysis of variance (ANOVA) with a Tukey post hoc test or a Kruskal-Wallis analysis with a post hoc Steel-Dwass or Steel test was used. A $p < 0.05$ was considered statistically significant. All statistical analyses were performed using JMP software (SAS Institute, Cary, NC, USA).

1.11. Ethical approval and consent to participate.
This study was reviewed and approved by the Central Ethics Review Board of the National Hospital Organization Headquarters in Japan (Tokyo, Japan) and Shinshu University (Nagano, Japan). The exact date when the ethical approval was obtained was August 17, 2019. The code number of the ethical approval was NHO H31-02. The authors attended educational lectures on medical ethics in 2020 and 2021, which were supervised by the Japanese government. The completion numbers for the authors are AP0000151756, AP0000151757, AP0000151769, and AP000351128. Consent to participate was required as this research was a clinical research. Subjects signed the informed consent when they were briefed on the clinical study and agreed with contents of clinical research. The authors attended a seminar on the ethics of experimental research using small animals on July 02, 2020 and July 20, 2021. They became familiar with the importance and ethics of animal experiments (National Hospital Organization Kyoto Medical Center and Shinshu University School of Medicine). The code number of the ethical approval for experiments with small animal was KMC R02-0702.

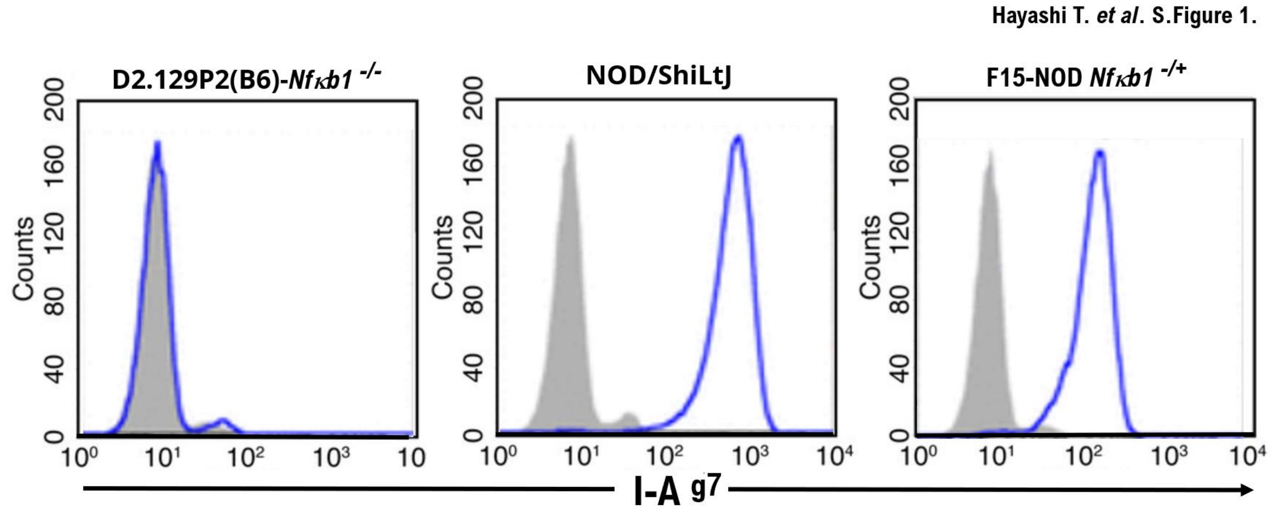

Figure S1. **The expression of class II MHC haplotype I-Ag7 on splenocytes obtained from F15 NOD *Nfκb1* heterozygote mice.** Fluorescence-activated cell sorter (FACS) analysis was used to assess the levels of antigen-presenting machinery by class II MHC on splenic B-cells as determined by gating on B220-positive cells in splenocytes derived from splenocytes of D2.129P2(B6)-*Nfkb1* homozygote (-/-) mice, NOD/ShiLtJ mice, and F15 NOD *Nfkb1* heterozygote (-/+) mice. B220 positive B cells were stained with anti–I-A^{g7} (blue lines), respectively, and then analyzed by FACS. The gray lines indicate the isotype control for each cell line. Histograms are representative of more than three experiments in which at least three mice were analyzed. Comparison of mean fluorescence intensities among analyzed samples demonstrated P < 0.05 for data represented. Experiments were conducted with 5 animals in each group.

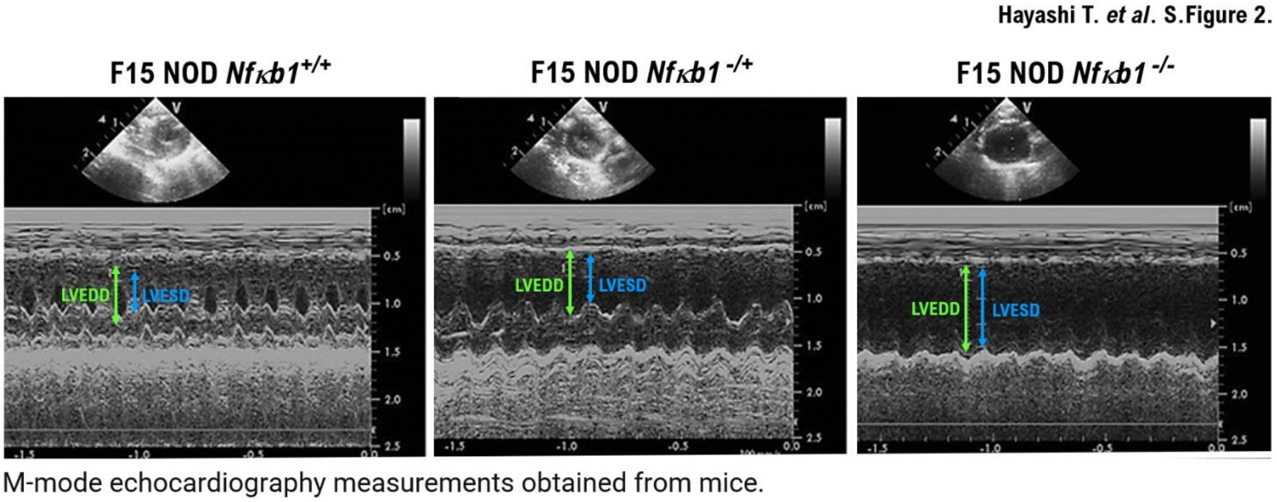

Figure S2. M-mode echocardiography examination for genetically modified mice. M-mode echocardiography measurements obtained from F15 NOD *Nfκb1* wild type (+/+) mice at 5 weeks of age, age matched F15 NOD *Nfκb1* heterozygote (-/+) mice, and F15 NOD *Nfκb1* homozygote (-/-) mice. LVEDD, left ventricle end-diastolic dimension; LVESD, left ventricle end-systolic dimension. Experiments were conducted with 20 animals in each group.

Hayashi T. *et al*. S.Figure 3.

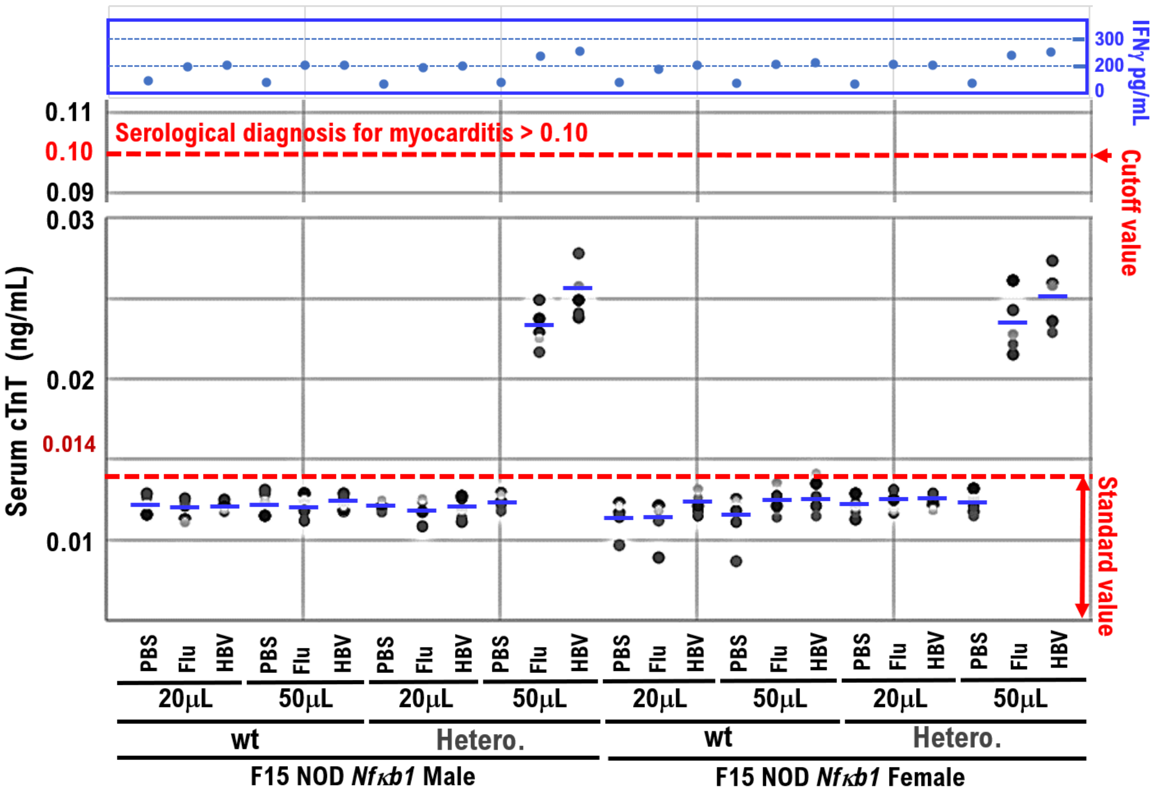

Figure S3. Measurement of serum cardiac troponin T (cTnT) in genetically modified mice. Increased serum cardiac troponin T (cTnT) concentration in F15-NOD *NfκB1* heterozygote male and female mice after high concentration (50 μL) of influenza HA vaccination or HBV vaccination. The two concentrations (20 μL, 50 μL) of PBS (as control), influenza HA vaccine, or HBV vaccine was inoculated at quadriceps femoris muscle of the left thigh muscle of 10-week-old F15 NOD *Nfκb1* wt male and female mice, and F15 NOD *Nfκb1* heterozygote male and female mice. Influenza HA vaccine or HBV vaccine (Bimmugen) was administered to mice by diluting the stock solution five-fold with PBS and inoculating 100 μL. In cases of F15-NOD *Nfκb1* heterozygote male mice and female mice inoculated with high concentration (50 μL) of influenza HA vaccine or HBV vaccine, the serum concentrations of cTnT were ≥0.02 ng/mL. However, in cases of F15-NOD *Nfκb1* heterozygote male mice and female mice inoculated with low concentration (20 μL) of influenza or HBV vaccine, the elevated serum concentrations of cTnT were not observed. No significance of gender difference was observed in the increase in serum cTnT concentration after each vaccination. The standard serum cTnT concentration is ≤0.014 ng/mL. In clinical practice, myocarditis is diagnosed when the serum cTnT concentration is ≥0.1 ng/mL. Each mouse group consisted of five mice. The serums were collected from each mouse, and then serum IFN-γ levels were measured by enzyme-linked immunosorbent assays (ELISAs) using a commercial kit (R&D Systems, Inc.) based on the manufacturer's instructions. In cases of serums derived from mice inoculated with influenza HA vaccine or HBV vaccine, the increase in serum IFN-γ levels were observed, particularly the concentrates of serum IFN-γ were significantly elevated in F15-NOD *Nfκb1* heterozygote male mice and female mice inoculated with high concentration (50 μL) of influenza HA vaccine or HBV vaccine. Experiments were conducted with 5 animals in each group.

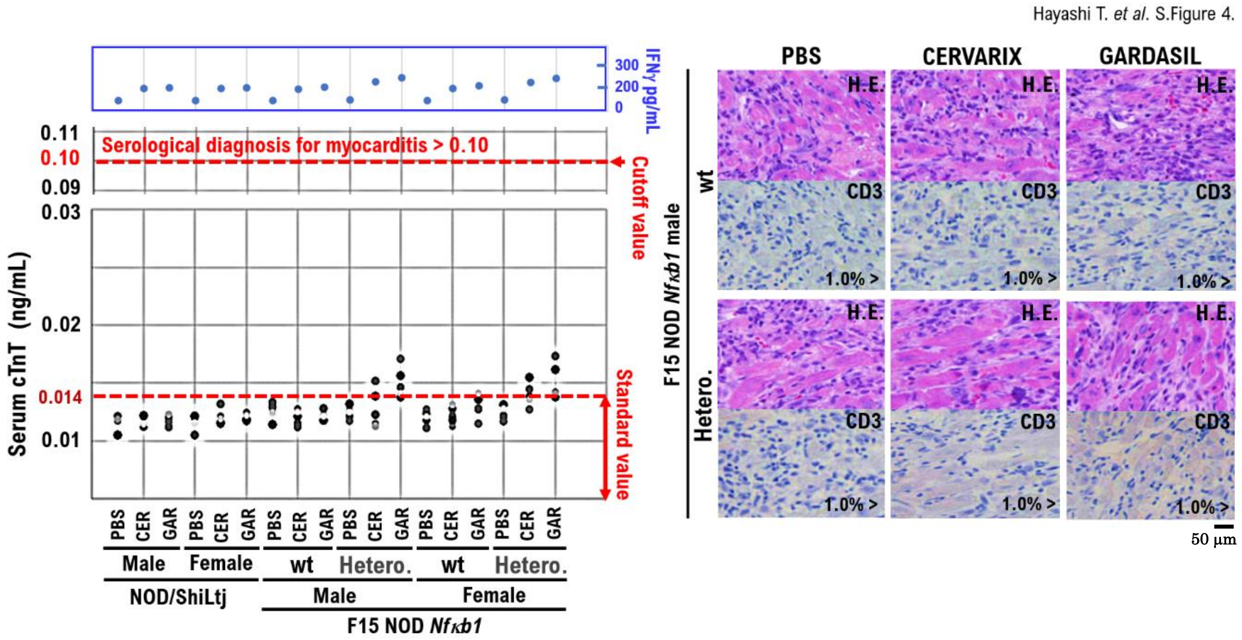

Figure S4. No onset of myocarditis in F15 NOD *Nfκb1* heterozygote mice after HPV vaccination. No observed onset of myocarditis in F15 NOD *Nfκb1* heterozygote mice after vaccination of CERVARIX or GARDASIL vaccine. **A** The concentration of serum cardiac troponin T (cTnT) in NOD/ShiLtj male and female mice, or F15-NOD *NfκB1* heterozygote male and female mice after intramuscular injection with 50 µL of CERVARIX (GlaxoSmithKline plc), 50 µL of GARDASIL (MSD K.K) vaccination, or PBS (control) were measured by mouse cTnT ELISA Kit (CUSABIO TECHNOLOGY LLC) according to the manufacturer's instructions. CERVARIX, GARDASIL or PBS were administered to mice by diluting the stock solution five-fold with PBS and inoculating 50 µL. Although in cases of NOD/ShiLtj male and female mice and F15 NOD *Nfκb1* heterozygote male and female mice inoculated with CERVARIX, GARDASIL or PBS, the serum concentration of cTnT was $\leq$ 0.02 ng/mL, the serum concentration of cTnT were slightly increased in F15 NOD *Nfκb1* heterozygote male and female mice inoculated with CERVARIX, GARDASIL. No gender difference was observed in the increase in serum cTnT concentration after each vaccination. The standard serum cTnT concentration is ≤0.014 ng/mL. In clinical practice, myocarditis is diagnosed when the serum cTnT concentration is ≥0.1 ng/mL. Each mouse group consisted of five mice. **B** Representative hematoxylin and eosin (H.E.)-stained tissue sections and immunohistochemically stained tissue sections with anti-CD3 monoclonal antibody of the hearts obtained from NOD/ShiLtj male and female mice, and F15 NOD *Nfκb1* wild type (wt) male and female mice, and F15 NOD *Nfκb1* heterozygote male and female mice. Infiltrations of CD3 positive T cells in myocardial tissues obtained from all tested mice are not observed. The finding supported that the onset of myocarditis is not observed in NOD/ShiLtj male and female mice, and F15 NOD *Nf κb1* heterozygote male and female mice, wild type male and female mice after intramuscular injection with 50 µL of CERVARIX (GlaxoSmithKline plc), 50 µL of GARDASIL (MSD K.K) or 50 µL of PBS. At 30 days after 1st dose immunization of CERVARIX, GARDASIL, or PBS, the mice received second doses of CERVARIX, GARDASIL or PBS. The blood and heart tissues were obtained at the time of dissection (Supplementary data excel file). All images x400. Experiments were conducted with 5 animals in each group.

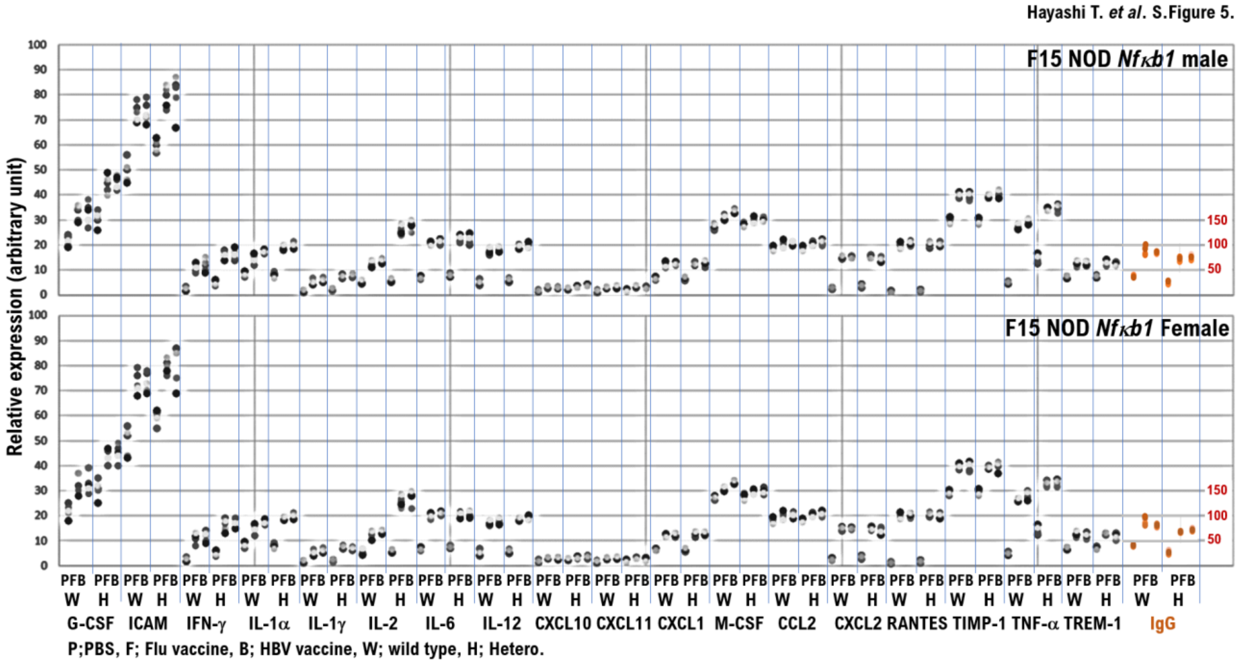

Figure S5. Elevated serum inflammatory cytokines in mouse serum derived from genetically modified mice. Elevated serum inflammatory cytokines in mouse serum derived from F15 NOD *Nfκb1* wild type mice and F15 NOD *Nfκb1* heterozygote mice after vaccination of influenza HA vaccine or HBV vaccine (Bimmugen). To collect serum for measuring of levels of serum inflammatory cytokines, mice were anesthetized with ether and bled retro-orbitally or alternatively at 3 days after immunizations of 50 μL of influenza HA vaccine or 50 μL of HBV vaccine (Bimmugen), or PBS. Cytokine and chemokine levels were measured in mouse serum (20× diluted) using the Mouse Cytokine Array Panel A (Catalog Number ARY006; R&D Systems, Inc.) based on the manufacturer's instructions. Levels of serum inflammatory cytokines derived from male mice (upper panel) and female mice (lower panel) are shown in this figure. Levels of serum inflammatory cytokines in mouse serum derived from F15 NOD *Nfκb1* wild type mice and F15 NOD *Nfκb1* heterozygote mice after vaccination are significantly elevated at 3 days after vaccination, but no elevation of inflammatory cytokines is observed in mouse serum derived from F15 NOD *Nfκb1* wild type mice and F15 NOD *Nfκb1* heterozygote mice after intramuscular injection of PBS as negative control. The levels of IgG, all listed inflammatory cytokine, which is markedly released from activated type 1 immunological responses, in male mice are more elevated compared to female mice. The levels of mouse IgG were measured in mouse serum using the Mouse IgG ELISA Kit (ab151276)(Catalog Number ab151276; Abcam plc. Cambridge Biomedical Campus, Cambridge, CB2 0AX, UK) based on the manufacturer's instructions. To collect serum for measuring of levels of serum cytokines, mice were anesthetized with ether and bled retro-orbitally or alternatively at 3 days after immunizations of 4 types of vaccines. The levels of mouse IgG were measured in mouse serum using the Mouse IgG ELISA Kit (Abcam :ab151276) based on the manufacturer's instructions. Experiments were conducted with 5 animals in each group.

Hayashi T. *et al*. S.Figure 6.

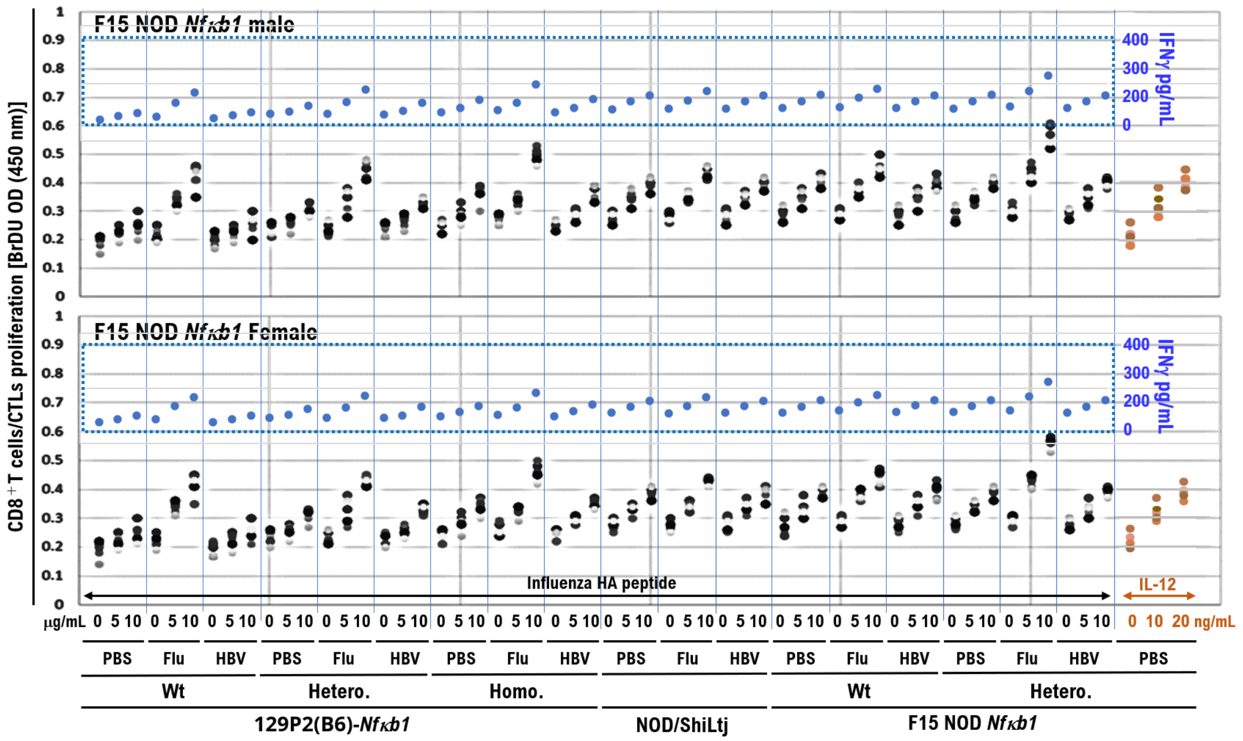

Figure S6. Proliferation of sub-purified CD8+ T cells under the coculture condition with Myosin heavy chain peptide. Activation of proliferation of sub-purified CD8+ T cells (also called as CTL) under the coculture condition with Myosin heavy chain peptide. Splenocytes from the mice immunized by intramuscular injection of 50 μL of influenza HA vaccine, 50 μL of HBV vaccine (Bimmugen) or PBS were harvested and depleted of red blood cells. Then, CD8+ T cells were sorted using a magnetic-activated cell sorting kit (CD8+ T Cell Isolation Kit II) according to the manufacturer's protocol. The isolated CD8+ T cells were resuspended in PBS containing 2% FBS, CD8+ T cells at a concentration of $1x10^6$ cells/ml were co-cultured with 100 μl of PBS containing a range of Influenza HA (46-54) Peptide (FMYSDFHFI) (M&S TechnoSystems, Inc.) (5 μg/mL or 10 μg/mL) (PeproTech) for a period of 48 hours. The cultured cells were harvested, and the proliferation of CD8+ T cells was evaluated using the BrdU Cell Proliferation ELISA kit (cat. no. ab126556; Abcam, Cambridge) based on the manufacturer's instructions. The supernatant was harvested and analyzed for the release of IFN-γ as internal control. After incubation, the culture supernatants were collected for cytokine detection. IFN-γ levels in the culture supernatants were measured by enzyme-linked immunosorbent assays (ELISAs) using a commercial kit (R&D Systems, Inc.) based on the manufacturer's instructions. In cases of coculture of Influenza HA (46-54) peptide with sub-purified CD8+ T cells derived from the mice inoculated with influenza HA vaccines, the activated proliferation of CD8+ T cells were markedly observed. In cases of coculture of Influenza HA (46-54) peptide with sub-purified CD8+ T cells derived from the mice inoculated with influenza HA vaccines, the increase in IFN-γ levels were markedly observed. Experiments were conducted with 5 animals in each group.

Hayashi T. *et al*. S.Figure 7.

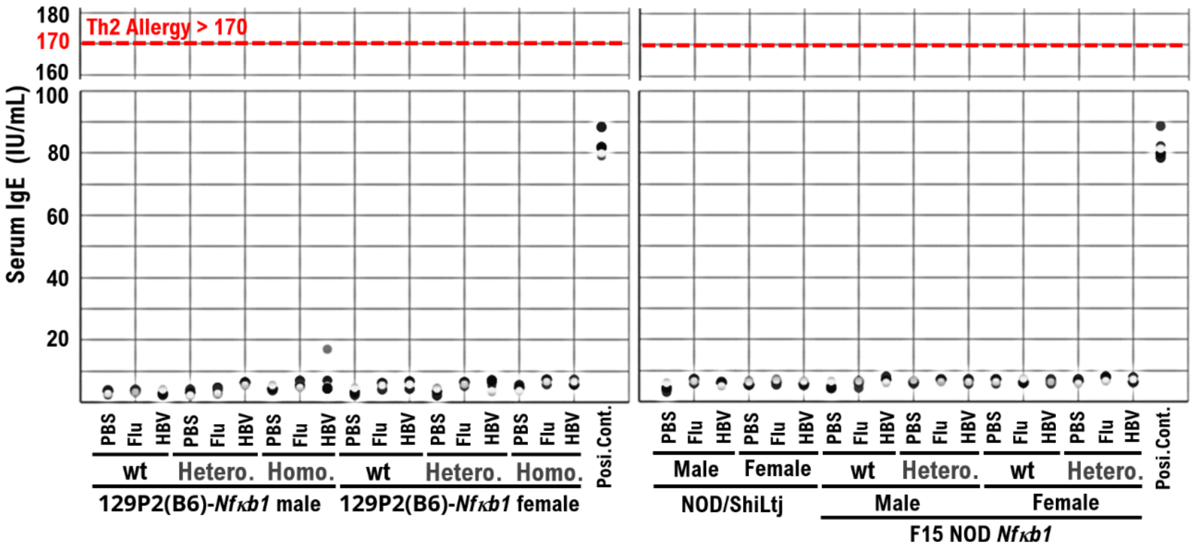

Figure S7. Allergy test for each genetically modified mouse after vaccination. No observed the increase in serum IgE in F15 NOD *Nfκb1* heterozygote mice after vaccination of influenza HA vaccine or HBV vaccine. The 50 µL of PBS (as control), 50 µL of influenza HA vaccine, or 50 µL of HBV vaccine was inoculated at quadriceps femoris muscle of the left thigh muscle of 10-week-old 129P2 (B6)-*Nfκb1* wild-type (wt), 129P2(B6)-*Nfκb1* heterozygote, 129P2(B6)-*Nfκb1* homozygote, NOD/ShiLtj, F15 NOD *Nfκb1* wt, and F15 NOD *Nfκb1* heterozygote mice. Influenza HA vaccine or HBV vaccine (Bimmugen) was administered to mice by diluting the stock solution five-fold with PBS and inoculating 50 µL. In NOD/ShiLtJ mice, 129P2 (B6)-*Nfκb1* homozygote mice or F15-NOD *Nfκb1* heterozygote mice inoculated with influenza or HBV vaccine, serum IgE levels were measured in mouse serum (10× diluted) using OpIEATM set mouse IgE (BD PharMingen, Lakes, NJ, USA) based on the manufacturer's instructions. The IgE levels in the serums derived from all tested mice were under the 10 IU/mL. Ten weeks-old BALB/c mice (Japan Clear, Meguro-ku, Tokyo, Japan) were intraperitoneally administered 100 µL of a solution containing Formalin treated Pseudomonas pertucinogena (FTPP) suspension (80 µg/Body) with Mite Extract DF (50 µg/Body) as experimental allergy model mouse, the mice were intraperitoneally second doses administered 100 µL of FTPP suspension and Mite Extract DF at 2 weeks after first injection. Serums were collected from each BALB/c mouse at 2 weeks after second doses injection. Although expression level of IgE is significantly elevated in the experimental allergy model mice, the research findings do not give the medical evidence suggesting that level of IgE as allergy response is induced in F15 NOD *Nfkb1* wild type mice and F15 NOD *Nfkb1* heterozygote mice. The standard value of IgE in adult is less than 170 IU/mL, probably IgE of babyhood at early stage is 5.0-10.0 IU/mL. Experiments were conducted with 5 animals in each group.

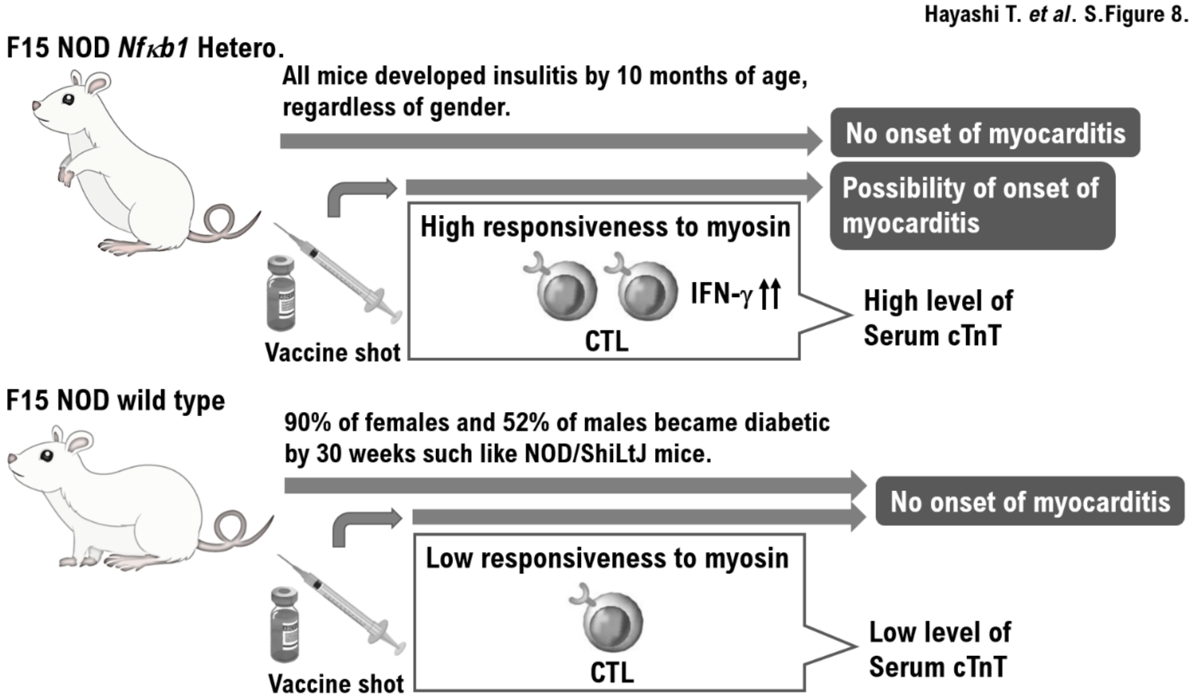

Figure S8. The onset of spontaneous myocarditis in F15 NOD *Nfκb1* heterozygote mice after vaccination. The onset of spontaneous myocarditis in F15 NOD *Nfκb1* heterozygote mice after vaccination with influenza HA vaccine or HBV vaccine (Bimmugen). All F15 NOD *Nfkb1* heterozygote mice developed insulitis by 10 months of age, regardless of gender. The 90% of females and 52% of males among F15 NOD *Nfkb1* wild type mice became diabetic by 30 weeks such like NOD/ShiLtJ mice. The onset of spontaneous myocarditis in F15 NOD *Nfκb1* heterozygote mice after vaccination with influenza HA vaccine or HBV vaccine (Bimmugen) was observed, but no observed onset of myocarditis in F15 NOD *Nfκb1* heterozygote mice after vaccination of CERVARIX or GARDASIL vaccine. High responsiveness to heart myosin by CD8-positive T cells (also called as Cytotoxic T cells) is markedly induced, and then high-level serum cTnT is detected in F15 NOD *Nfκb1* heterozygote mice after vaccination with influenza HA vaccine or HBV vaccine (Bimmugen). No onset of spontaneous myocarditis among F15 NOD *Nfkb1* wild type mice is observed after vaccination with influenza HA vaccine or HBV vaccine (Bimmugen). The expression of *Nfκb1* gene is significance for the onset of spontaneous myocarditis by vaccination with influenza HA vaccine or HBV vaccine (Bimmugen).
